# Gene mutations associated with early onset familial Alzheimer’s disease in China: An overview and current status

**DOI:** 10.1002/mgg3.1443

**Published:** 2020-08-06

**Authors:** Qi Qin, Yunsi Yin, Yan Wang, Yuanyuan Lu, Yi Tang, Jianping Jia

**Affiliations:** ^1^ Innovation Center for Neurological Disorders Department of Neurology Xuanwu Hospital Capital Medical University Beijing China; ^2^ Beijing Key Laboratory of Geriatric Cognitive Disorders Beijing China; ^3^ Clinical Center for Neurodegenerative Disease and Memory Impairment Capital Medical University Beijing China; ^4^ Center of Alzheimer's Disease Beijing Institute for Brain Disorders Beijing China

**Keywords:** amyloid precursor protein, early‐onset familial Alzheimer's disease, presenilin 1, presenilin 2

## Abstract

**Background:**

Mutations of three causative genes, namely presenilin 1 (*PSEN1*), presenilin 2 (*PSEN2*), and amyloid precursor protein (*APP*), have been identified as the major causes of early‐onset familial Alzheimer's disease (EOFAD). The prevalence of causative gene mutations in patients with EOFAD has been reported in previous studies worldwide but remains unclear in China. The patients with these known mutations always show considerable clinical phenotypic variability. However, to date, there have been no detailed descriptions of the clinical phenotypes associated with these Chinese EOFAD mutations. Thus, the aim of this study was to describe all of the known mutations in three EOFAD causative genes and genotype–phenotype correlations in Chinese patients with EOFAD.

**Method:**

We systematically searched the PubMed, MEDLINE, CNKI, VIP, and WAN‐FANG databases to find Chinese EOFAD mutations in reports from inception through May 2020.

**Result:**

We identified 31 studies reporting mutations of three causative genes in China. 10 mutations in *APP* gene, 27 mutations in *PSEN1* gene and six mutations in *PSEN*2 were discovered in Chinese EOFAD. This review summarized all these probably pathogenic mutations as well as its clinical features. To the best of our knowledge, this is the first systemic review of causative gene mutations in patients with EOFAD in China.

**Conclusion:**

The analysis of the genetic and clinical phenotype correlations in this review supports the idea that the clinical phenotype might be influenced by specific genetic defects. It also suggests genetic testing and genotype–phenotype correlations are important for the accurate diagnosis and for understanding disease‐associated pathways and might also improve disease therapy and prevention.

## Abstract 2

This review summarizes all probably pathogenic mutations reported in China in APP, PSEN1, and PSEN2, as well as its clinical features. The analysis of the genetic and clinical phenotype correlations in this review supports the idea that the clinical phenotype might be influenced by specific genetic defects. It also suggests genetic testing and genotype–phenotype correlations are important for the accurate diagnosis and for understanding disease‐associated pathways and might also improve disease therapy and prevention.

## 1 BACKGROUND

Alzheimer's disease (AD) is the most common form of dementia and a major public health problem in the world. Currently, there are more than 44 million dementia cases worldwide, with that number is predicted to more than triple by 2050 (Lane, Hardy, & Schott, 2018; Wu et al., 2012). Health care costs of dementia in 2015 surpassed $818 billion (USD), and this figure is estimated to be as high as $2 trillion by 2030 (Alzheimer's, 2016). In China, the number of patients with AD accounts for approximately 25% of the entire population with AD worldwide (Jia et al., 2020) and the total cost of AD was $167.74 billion per year, accounting for 1.47% of the gross domestic product (GDP) (Jia et al., 2014, 2018), creating a huge challenge for policymakers, healthcare professionals, AD patients, and family members.

The typical clinical pattern of AD starts with episodic memory dysfunction then progresses to loss of cognitive functions (Ballard et al., 2011). The main hallmarks of AD are the accumulation of amyloid beta (Aβ) protein in the form of senile plaques and neurofibrillary tangles of hyperphosphorylated tau protein. AD is a multifactorial and complex disease, and several genetic and/or environmental factors can contribute to disease progression (Alzheimer's, 2016). According to the age of onset of the disease, two types of AD are distinguished: early‐onset AD (EOAD) and late‐onset AD (LOAD) (Reitz & Mayeux, 2014). LOAD, also called sporadic AD, is the most common type of AD with an age of onset later than 65 years (Mukherjee et al., 2019). The ε4 allele of the *APOE* gene is the major risk factor for pathogenesis of LOAD (Mahoney‐Sanchez, Belaidi, Bush, & Ayton, 2016; Yu, Tan, & Hardy, 2014). EOAD is commonly diagnosed with an onset age of earlier than 65 years and accounts for 5%–10% of all AD cases (Tellechea et al., 2018; Zhu et al., 2015). Approximately 10% of EOAD patients displays an autosomal‐dominant pattern of inheritance (Dai, Zheng, Zeng, & Zhang, 2018). Three main genes are involved in early‐onset familial AD (EOFAD), *APP* (HGNC: 620, OMIM: 104760), *PSEN1* (HGNC: 9508, OMIM: 104311), and *PSEN2* (HGNC: 9509, OMIM: 600759), encoding amyloid precursor protein, presenilin‐1, and presenilin‐2, respectively (Mendez, 2019). Mutations in all these three genes could result in enhanced Aβ production and deposition (Raux et al., 2005). To date, there are more than 400 known mutations of these three genes that result in EOFAD, and *PSEN1* mutations are responsible for approximately 75% of genotyped families positive for a mutation, whereas *APP* and *PSEN2* mutations account for 13% and 12%, respectively (https://www.alzforum.org/mutations). The prevalence of these causative gene mutations in patients with EOFAD has been reported in previous studies, but it remains unclear in China (Giri, Zhang, & Lu, 2016). The patients with these known mutations always show considerable clinical phenotypic variability (Shao, Peng, & Wang, 2017; Zou, Liu, Che, & Huang, 2014). However, to date, there have been no detailed descriptions of the clinical phenotypes associated with these Chinese EOFAD mutations (Pei, Giron, Jia, & Wang, 2014). Thus, we performed a systemic review for these three genes in this study to characterize the mutation spectrum and describe the genotype–phenotype correlations in patients with EOFAD in China which may provide further clues to improve our understanding and future treatment of AD.

## 2 MATERIALS AND METHODS

### 2.1 Editorial policies and ethical considerations

This study received approval from the institutional review board at Xuan Wu Hospital Capital Medical University.

### 2.2 Study selection

We searched PubMed, MEDLINE, CNKI, VIP, and WAN‐FANG databases to find Chinese EOFAD mutations in reports published till May 2020 using the terms: “early‐onset AD,” “China,” “APP,” “PSEN1,” and “PSEN2”; all identified articles published in English and Chinese, articles referenced therein were reviewed. The GenBank reference sequence and version number of all mutations are listed in Result Section. The mutation nomenclature followed the HGVS nomenclature guidelines (http://www.hgvs.org/mutnomen/).

### 2.3 Computational predictive programs

The SIFT (https://sift.bii.a‐star.edu.sg/www/SIFT_seq_submit2.html) and PolyPhen‐2 prediction software (http://genetics.bwh.harvard.edu/pph2/index.shtml) were used to predict the pathogenicity of variants. PolyPhen‐2 can evaluate a mutation qualitatively, as benign, possibly damaging or probably damaging.

## 3 RESULT

### 3.1 EOFAD susceptibility gene mutations in China

*APP*,* PSEN1*, and *PSEN2* are the known genes that mutated to cause EOFAD. These mutations are only responsible for 30%–50% of autosomal dominant AD cases and about 0.5% of AD in general, however, they are important for the presymptomatic diagnostics of patients from EOFAD families (Wu et al., 2012). In addition, the characterization of these genes and mutations can advance our understanding on the molecular mechanism of AD (Shao et al., 2017). To date, approximately 302 different pathogenic mutations in *PSEN1*, 51 mutations in *PSEN2* and 54 mutations (or gene duplication) in *APP* have been identified in EOFAD (https://www.alzforum.org/mutations). In China, the EOFAD gene mutation research has lagged behind for a long time, only a few gene mutations have been reported.

### 3.2 The discovery history of EOFAD causative genes

The high incidence of AD‐like clinical and neuropathological changes in older patients with Down syndrome (trisomy 21) led to the idea that the causative gene of familial AD may be located on chromosome 21 (Potter, Granic, & Caneus, 2016; Prasher et al., 1998). In 1987, a gene locus at 21q11.2 to 21q22.2 was found by genetic linkage study (Goldgaber, Lerman, McBride, Saffiotti, & Gajdusek, 1987). In 1991, the first missense mutation (Val‐Ile) in *APP* was reported on chromosome 21p2 in one single EOFAD family, thus providing the first possible association between the *APP* mutations and abnormalities in amyloid processing (Goate et al., 1991). Since several families with early onset AD had no linkage to chromosome 21, Goate (2006), suggested that genetic heterogeneity might exist within EOFAD. Genetic heterogeneity was further supported by several independent studies when a locus for EOFAD on 14q24 was identified in 1992. In 1995, the *PSEN1* gene, which encodes the protein presenilin 1 required for γ‐secretase to produce amyloid‐beta (Aβ) from APP, was cloned for the first time and was identified as one of pathogenic genes for the EOFAD (Perez‐Tur et al., 1995). In the same year, the missense mutation in *PSEN2* was found on the long arm of chromosome 1 in two different studies (Rogaev et al., 1995). Since then, more than 400 mutations of EOFAD causative genes have been found. The majority of pathogenic mutations were found in *PSEN1* gene. Mutations in *APP* and *PSEN2* are quite rare, but are possible causative factors for EOFAD.

The genetic background of Chinese AD patients was not well characterized. According to the most recent study about EOAD discovered in Asian countries, only four mutations in *APP* gene, eight mutations in *PSEN1* gene, and five mutations in *PSEN*2 were discovered in Chinese EOFAD (Bagyinszky, Youn, An, & Kim, 2016). However, several recent studies reported additional novel mutations. Most of the variants, discovered in Chinese families and patients, are novel mutations, which have not been described in any other population.

### 3.3 Mutations in APP

The gene encoding APP is located on chromosome 21q21.3. APP is a transmembrane protein, which can play a role in synaptic plasticity (Turner, O'Connor, Tate, & Abraham, 2003). Three enzymes, the α‐, β‐, and γ‐secretases, have cleavage sites in APP protein. Proteolysis of APP by α‐ and γ‐secretases results in nonpathogenic fragments (sAPPα and α‐C‐terminal fragment) in nonamyloidogenic pathway (Yoshikai, Sasaki, Doh‐ura, Furuya, & Sakaki, 1991). In the amyloidogenic pathway, abnormal cleavage of APP by β‐ and γ‐secretase could result in two major Aβ species that include sAPPβ and β‐CTF (Thinakaran & Koo, 2008). The action of γ‐secretases on β‐CTF generates Aβ, which forms a key component of amyloid plaques in AD brain. It was suggested that Aβ peptide may be important in synaptic vesicle regulation, but the oligomer form of peptide can be involved in neurotoxicity. The mutations of *APP* could alter the γ‐secretase function with increased Aβ42/Aβ40 ratio by increasing Aβ42 levels and decreasing Aβ40 levels. Aβ42 and Aβ40 peptides and Aβ42/40 ratio have been established as the most important biomarkers for AD. To date, *APP* mutations are known to cause AD. The following ten *APP* mutations have been found in Chinese patients (Table 1).

**Table 1:** Mutations of APP discovered in China

Gene	Protein change	Location in the gene (exon)	Gene mutation	Location in APP protein	Was it discovered before?	Functional data	PolyPhen Scores	SIFT scores	Reference
APP	Asp678His	16	c.2032G> C	N‐terminal	No	Increased Aβ production, Aβ 42/40 ratio and prolong the Aβ 42 oligomer state	0.62 (possible damaging)	1 (tolerated)	Huang et al. (2019)
	Lys687Gln	16	c.2059A>C	N‐terminal	No	Not available	0.99 (probably damaging)	0 (damaging)	Jiang et al. (2019)
	Val710Gly	17	c.2129T>G	TM‐1	No	Not available	1 (probably damaging)	0 (damaging)	Thajeb et al. (2009)
	Val715Met	17	c.2143G>A	TM‐1	Yes, ”French APP"	Destroy the Aβ40 cleavage site of γ‐secretase	0.99 (probably damaging)	0 (damaging)	Nan et al. (2011) (Nan et al. (2014)
	Ile716Thr	17	c.2147T>C	TM‐1	Yes, in Italy	Increase γ‐secretase cleavage at position 42 or 43	0.99 (probably damaging)	0 (damaging)	Wang, Qin, et al. (2017)
	Val717Ile	17	c.2149G>A	TM‐1	Yes, "London APP"	Increased Aβ42/40 ratio and decreased Aβ40 in CHO and HEK293	0.99 (probably damaging)	0 (damaging)	Zhang et al. (2017)
	IIe718Leu	17	c.2150T>C	TM‐1	No	Not available	0.77 (possible damaging)	0.1 (tolerated)	Thajeb et al. (2009)
	Leu720Ser	1**7**	c.2159T>C	TM‐1	No	Not available	1 (probably damaging)	0 (damaging)	Thajeb et al. (2009)
	Met722Lys	17	c.2166T>A	TM‐1	No	1.7‐fold increased Aβ42/40 in N2a cells	1 (probably damaging)	0 (damaging)	Wang et al. (2015)
	Lys724Met	17	c.2171A>T	C‐terminal	No	2.23‐fold increased Aβ42/40 in HEK293 cell	0.98 (probably damaging)	0.01 (damaging)	Peng et al. (2014)

The novel Asp678His (NM_000484.4:c.2032G>C, NP_000475.1:p.Asp678His) *APP* mutation, also named “Taiwan mutation,” was detected in all ten symptomatic patients with EOFAD and the seven asymptomatic family members (Huang et al., 2019). Sequencing showed a G → C nucleotide substitution in the *APP* that resulted in an aspartate to histidine mutation at the 7th position of Aβ. Cellular and biochemical analyses have revealed that this mutation can increase Aβ production, Aβ 42/40 ratio, and prolong the Aβ 42 oligomer state (Chen et al., 2012).

A novel variant Lys687Gln (NM_000484.4:c.2059A>C, NP_000475.1:p.Lys687Gln) was found in two Chinese family by whole‐exome sequencing performed in 15 Chinese patients with FAD (Jiang et al., 2019). It was an A → C missense mutation resulting in a lysine to glutamine substitution at codon 687. According to the PolyPhen‐2 and SIFT Mutation Taster prediction software, they all suggested this mutation was considered to be probably pathogenic.

Val715Met (NM_000484.4:c.2143G>A, NP_000475.1:p.Val715Met) mutation was revealed in c.2143 G>A in exon 17 of APP. It was reported in Chinese families in 2011 (Nan, Han, Fan, & Chen, 2011) and 2014 (Nan, Han, Fan, & Chen, 2014). Because the mutation was first discovered in a French family with progressive memory decline, the mutation is also called “French APP” (Ancolio et al., 1999). Val715Met was expressed in human embryonic kidney 293 (HEK293) cell lines, and a significant decrease in Aβ40 levels (2‐fold) was observed. Aβ42 levels did not change, but the ratio of Aβ42/Aβ40 increased (1.8‐fold). These findings suggested that this mutation might destroy the Aβ40 cleavage site of gamma‐secretase (Park, Na, Lee, Kim, & Ki, 2008).

Ile716Thr (NM_000484.4:c.2147T>C, NP_000475.1:p.Ile716Thr) was a mutation of c.2147T>C in exon 17 of *APP* gene (Wang, Qin, et al., 2017). Previous studies have identified a mutation at the same codon resulting in an Ile‐to‐Val substitution (I716V) (Eckman et al., 1997). Mutations at codons 716 increase the proportion of β‐secretase cleavage at position 42 or 43 in the Aβ sequence. Suarez‐Calvet found that I716T increased Aβ42 and decreased Aβ40 secretion in the conditioned medium compared to wtAPP‐expressing cells. And Aβ38 was also significantly increased. APP‐I716T produced a dramatic increase in the Aβ42/40 ratio and decreased the Aβ40/Aβ38 ratio. The mechanism seems to be a change in the product line preference of γ‐secretase toward the Aβ38 line (Eckman et al., 1997; Suarez‐Calvet et al., 2014). Cells transfected with cDNAs bearing this mutation produce more Aβ 1‐42 (43) than those transfected with wild‐type APP (Eckman et al., 1997; Forloni et al., 2002).

Val717Ile (NM_000484.4:c.2149G>A, NP_000475.1:p.Val717Ile, “London APP”) is one of the most common APP mutations worldwide (Brooks et al., 1995; Dai et al., 2018; Talarico et al., 2010; Thajeb, Wang, Chien, & Harrigan, 2009; Wang et al., 2015; Xia et al., 2015) and was reported in five Han Chinese families with EOFAD (Zhang, Xie, Wang, Feng, & Jia, 2017). The missense mutation of G to A (c.2149G>A) resulted in a valine to isoleucine substitution at codon position 717 in exon 17 of APP. This mutation was analyzed in several cell lines (Brooks et al., 1995; Kennedy et al., 1993; Talarico et al., 2010; Tamaoka et al., 1994). CHO and HEK293 experiments suggested that Val717Ile increases the Aβ42/40 ratio (Tamaoka et al., 1994). Decreased Aβ40 levels were reported after analyzing this mutation in HEK293, COS cell (Talarico et al., 2010).

The mutations causing isoleucine to leucine substitution at codon 718 of *APP* (IIe718Leu, NM_000484.4:c.2150T>C, NP_000475.1:p.IIe718Leu), leucine to serine substitution at codon 720 (Leu720Ser, NM_000484.4:c.2159T>C, NP_000475.1:p.Leu720Ser), and valine to glycine substitution at codon 710 (Val710Gly, NM_000484.4:c.2129T>G, NP_000475.1:p.Val710Gly) were also reported in Chinese/Taiwanese patients with early onset familial AD (Thajeb et al., 2009). However, no functional data were reported in these mutations.

Met722Lys (NM_000484.4:c.2166T>A, NP_000475.1:p.Met722Lys) causes a novel missense mutation by ATG conversion to AAG at codon position 722 in exon 17 of the *APP* gene in a Chinese EOFAD pedigree in 2015 (Wang et al., 2015). The expression of APP M722K in mouse neuroblastoma N2a cells induced a 1.7‐fold increased ratio of Aβ42 to Aβ40 without changes in sAPPα and sAPPβ. Tau phosphorylation was also increased.

Lys724Met (NM_000484.4:c.2171A>T, NP_000475.1:p.Lys724Met) missense mutation located at position 724 in exon 17 of *APP*, causing an A/T amino acid substitution (Peng et al., 2014). It was identified in 2014 in China by using genetic analysis of three collected patients’ DNA samples. When APP695 with K724M mutation was ectopically expressed in HEK293 cell, the ratio of Aβ42 to Aβ40 was 2.23‐fold higher than that of wild‐type control.

### 3.4 Mutations in PSEN1

*PSEN1* gene is located on chromosome 14q24.3, and it is a vital component of the γ‐secretase complex, which cleaves APP into Aβ fragments (Steiner, Fluhrer, & Haass, 2008). PSEN1 is localized primarily to the endoplasmic reticulum and helps in protein processing (Bezprozvanny & Mattson, 2008). Mutations in *PSEN1* account for up to 50% of EOAD, with complete penetrance and early age of onset (Giri et al., 2016). Mutant γ‐secretase increases Aβ42 level while it decreases Aβ40 level, leading to an increase in the Aβ42/40 ratio. Morphologic variants in Aβ plaques due to *PSEN1* mutations may result in cotton wool plaques (Crook et al., 1998; Miki et al., 2019). Cotton wool plaque is formed by large rounded Aβ deposits, and it tends to be immune‐positive for Aβ42 rather than for Aβ40 (Zhang et al., 2015). The majority of *PSEN1* mutations are missense mutations, but a few insertion and deletions have also been detected. In addition to their role in γ‐secretase activity, *PSEN1* mutations may compromise neuronal function, affecting GSK‐3β activity and kinesin‐I‐based motility, thus leading to neurodegeneration (Giri et al., 2016). To date, 295 pathogenic mutations have been identified in *PSEN1*, of which 70% mutations occur in exons 5, 6, 7, and 8. Twenty‐seven mutations were reported in China till now (Table 2).

**Table 2:** Mutations of PSEN1 discovered in China

Gene	Protein change	Location in the gene (exon)	Gene mutation	Location in PS1 protein	Was it discovered before?	Functional data	PolyPhen Scores	SIFT scores	Reference
PSEN1	Val97Leu	4	c.289G>T	TM‐1	No	Higher β‐secretase activity in human neuroblastoma cells	0.99 (probably damaging)	0 (damaging)	Jia et al. (2005)
	Val103Gly	4	c.308T>G	HL‐1	No	Not available	0.999 (probably damaging)	0 (damaging)	Gao et al. (2019)
	Phe105Cys	4	c.314T>G	HL‐1	No	Not available	0.99 (probably damaging)	0 (damaging)	Jiao et al. (2014)
	Phe105Leu	4	c.313T>C	HL‐1	Yes, in Germany	Not available	0.978 (probably damaging)	0.26 (tolerated)	Wei et al. (2018)
	Gly111Val	4	c.332G>T	HL‐1	No	1.8‐fold increase in Aβ42/40 ratio in HEK293	0.99 (probably damaging)	0 (damaging)	Qiu et al. (2020)
	Glu116Lys	5	c.346G>A	HL‐1	Yes	Not available	0.99 (probably damaging)	0 (damaging)	Li et al. (2016)
	Ala136Gly	5	c.407C>G	HL‐1	No	Survival of mutant neuroblastoma cells	0.98 (probably damaging)	0.09 (tolerated)	Xu et al. (2002)
	Met139Leu	5	c.415A>T	TM‐II	No	Not available	0.96 (probably damaging)	0 (damaging)	Qiu et al. (2019)
	His163Arg	6	c.488A>G	HL‐2	Yes	Increased Aβ42	0.84 (possibly damaging)	0.1 (tolerated)	Shi et al. (2015)
	Ile167del	6	c.497‐499delTTA	TM‐III	No	Not available	Not available	Not available	Jiao et al. (2014)
	Ser169del	6	c.507‐509delATC	TM‐III	No	Disturbances in posttranslational modifications	Not available	Not available	Guo et al. (2010)
	Phe177Val	6	c.529T>G	TM‐III	No	Not available	0.84 (probably damaging)	0 (damaging)	Gao et al. (2019)
	Ile202Phe	7	c. 604A>T	TM‐IV	No	Not available	0.978 (probably damaging)	0.05 (tolerated)	Li, Yang, et al. (2019)
	Gly206Val	7	c.617G>T	TM‐IV	Yes, in the USA	Not available	1 (probably damaging)	0 (damaging)	Li, Yang, et al. (2019)
	His214Arg	7	c.641A>G	HL‐IV	No	Not available	0.999 (probably damaging)	0 (damaging)	Li, Yang, et al. (2019)
	Gln222leu	5	c.665A>T	TM‐V	No	Not available	0.997 (probably damaging)	0.01(damaging)	Wang et al. (2019)
	Leu226Arg	7	c.677T>G	TM‐V	Yes	Affect transmembrane domain of PSEN1	1 (probably damaging)	0 (damaging)	Ma et al. (2019)
	Met233Leu	7	c.697A>C	TM‐V	Yes, in France and Australia	Elevated (3.2‐fold) Aβ42/40 ratio in CHO	0.97 (probably damaging)	0.04 (damaging)	Jiang et al. (2015)
	Leu248Pro	7	c.743T>C	TM‐VI	No	Not available	1 (probably damaging)	0.09 (tolerated)	Jiao et al. (2014)
	Ile249Leu	7	c.745A>C	TM‐VI	No	Increased Aβ42, increased Aβ42/40 ratio	0.994 (probably damaging)	0.23 (tolerated)	Shen et al. (2019)
	Tyr256Asn	7	c.766T>A	TM‐VI	No	Not available	0.999 (probably damaging)	Not available	Li, Yang, et al. (2019)
	Arg352Cys	10	c.1054C>T	HL‐VI(b)	No	Not available	0.92 (probably damaging)	0.03 (damaging)	Jiang et al. (2015)
	Gly378Glu	11	c.1133G>A	TM‐VIII	Yes, in France	3.2‐fold increase in Aβ42/40 ratio in HEK293	1 (probably damaging)	0 (damaging)	Cao et al. (2014), Wang, Hao, et al. (2017)
	Phe386Ile	11	c.1156T>A	TM‐VII	Yes, in France and Japan	Increased Aβ42, decreased Aβ40, decreased Aβ42/38	0.999 (probably damaging)	0.03 (damaging)	Shea et al. (2017)
	Phe388Leu	11	c.1164C>G	TM‐VII	No	Increased Aβ42 secretion, increased Aβ42/40 ratio	0.99 (probably damaging)	0 (damaging)	Zhan et al. (2017)
	Pro433Ser	12	c.1297C>T	HL‐VIII	No	Increased Aβ42, Increased Aβ43, increased Aβ42/40 ratio	0.999 (probably damaging)	0 (damaging)	Shen et al. (2019)
	Ala434Thr	12	c.1300G>A	HL‐VIII	No	Not available	0.99 (probably damaging)	0 (damaging)	Jiao et al. (2014)

Val97Leu (NM_000021.4:c.289G>T, NP_000012.1:p.Val97Leu) was described for the first time in a Chinese family with EOAD in 2005 by Dr. Jia's group (Jia et al., 2005). To validate the pathogenic nature of this mutation, *PSEN1* Val97Leu was first expressed in human neuroblastoma (SH‐SY5Y) cells, and the Aβ concentration was monitored with ELISA and radioimmunity methods. The data showed that intracellular and extracellular Aβ production was higher in the mutant cells, suggesting that the mutation is pathogenic. In the following year, the same group also examined levels of Aβ precursor protein cleaving enzyme (BACE) and amyloid precursor protein to explore their impact upon Aβ production. Dr. Jia group results revealed that the presenilin‐1 V97L variant could elevate both intracellular and extracellular Aβ42 concentration. However, the total Aβ did not alter, consistent with unchanged BACE and APP expression levels (Fang, Jia, & Jia, 2006). In the year 2012, Dr. Jia's group expressed human Val97Leu mutant presenilin‐1 in transgenic mice and found that the mutant presenilin‐1 induced spatial memory deficit and tau hyperphosphorylation. Furthermore, in our in vitro N2a cells studies, we found that the tau hyperphosphorylation caused by the overexpression of the mutant PS1 protein depends upon GSK‐3 activity and is likely to be mediated through the PI3K/Akt/GSK‐3 intracellular pathway (Wang, Cheng, Qin, & Jia, 2012).

Dr. Jia's group also identified a novel Gly111Val (NM_000021.4:c.332G>T, NP_000012.1:p.Gly111Val) mutation in a male patient, which resulted in a glycine to valine substitution at codon 111. In vitro, this mutation elevated the Aβ42/Aβ40 ratio 1.8 times, by reducing Aβ40 concentration while no significantly increasing was found in Aβ42 concentration (Qiu et al., 2020). PolyPhen‐2 and SIFT Mutation Taster also suggested that Gly111Val was probably pathogenic.

In 2016, a novel heterozygous mutation, Glu116Lys (NM_000021.4:c.346G>A, NP_000012.1:p.Glu116Lys), was identified in an EOFAD family. This G to A mutation located in exon 5 of *PSEN1*, leading to a glutamic to lysine substitution (Li et al., 2016). No experiments were performed to establish the pathogenic nature of the mutation. Based on PolyPhen‐2 and SIFT Mutation Taster prediction software, this mutation was suggested to be probably pathogenic.

Ala136Gly (NM_000021.4:c.407C>G, NP_000012.1:p.Ala136Gly) was discovered in a Chinese family. Sequencing indicated a GCT to GGT missense mutation in code 136 of exon 5, resulting in a substitution from alanine to glycine. When this variant was expressed in human neuroblastoma cells, the survival of the mutant cells decreased significantly, suggesting that this mutation could have deleterious effects (Xu, Jia, & Sun, 2002).

A novel pathogenic variant *PSEN1* Met139Leu (NM_000021.4:c.415A>T, NP_000012.1:p.Met139Leu) was located at helix of PSEN1 transmembrane. In vitro studies revealed that Met139Leu reduced the Aβ40 levels while the promotion of Aβ42 levels was no significant. The Aβ42/40 ratios of Met139Leu were elevated for 1.6 times in HEK293‐APP cells, which indicated M139L was probably pathogenic. But no changes were found in tau phosphorylation (Qiu et al., 2019).

The pathogenic *PSEN1* His163Arg mutation (NM_000021.4:c.488A>G, NP_000012.1:p.His163Arg) was identified in a clinical EOAD patient with an autosomal dominant family history (Shi et al., 2015). This residue is on exon 6, hydrophilic loop II, a conserved domain in *PSEN2* His169. This common pathogenic mutation has been reported from the beginning of 1995 in at least 10 families. It suggested that this mutation could increase the level of Aβ42 (Qi, Morishima‐Kawashima, Sato, Mitsumori, & Ihara, 2003).

Ser169del (NM_000021.4:c.507‐509delATC, NP_000012.1:p.Ser169del) was described in a Chinese family with EOAD in 2009. This variant showed trinucleotide deletion in coding region predicting a deletion of one amino acid. Two additional mutations, Ser169Pro and Ser169Leu, which are involved in EOAD with rapid disease progression, were also described for codon 169. Ser169del might be associated with disturbances in posttranslational modifications, in the protein structure or in the interactions with other proteins because of the missing–OH group (Guo et al., 2010).

Phe105Cys (NM_000021.4:c.314T>G, NP_000012.1:p.Phe105Cys), Leu248Pro (NM_000021.4:c.743T>C, NP_000012.1:p.Leu248Pro), Ile167del (NM_000021.4:c.497‐499delTTA, NP_000012.1:p.Ile167del), and Ala434Thr (NM_000021.4:c.1300G>A, NP_000012.1:p.Ala434Thr) were observed with the disease phenotype in four families in 2014 by Jiao (Jiao et al., 2014). Although no more functional data were shown, pathogenicity predictions revealed that these four mutations were damaging using SIFT software. In addition, according to the algorithm reported from Guerreiro RJ, they predicted that I167del, A434T, and F105C were classified as definitely pathogenic, whereas the L248P mutation was considered to be probable pathogenic. Another mutations at codons 105, Phe105Leu (c.313T>C, p.F105L) has also been found in a Chinese EOFAD family. However, these mutation were lack of functional data (Wei et al., 2018).

In 2019, Dr. Gao performed a genetic screening of *APP*, *PSEN1*, and *PSEN2* hot spots in 148 index patients with independent family histories including EOAD and LOAD identified at multiple centers. Among *PSEN1* mutations, Val103Gly (NM_000021.4:c.308T>G, NP_000012.1:p.Val103Gly) and Phe177Val (NM_000021.4:c.529T>G, NP_000012.1:p.Phe177Val) are here first recognized as probably pathogenic with structural implications. Two variants likely decrease interactions with other hydrophobic residues in γ‐secretase and cause structural impairment (Gao et al., 2019).

Ile202Phe (NM_000021.4:c. 604A>T, NP_000012.1:p.Ile202Phe) was first reported in a Chinese EOFAD family. I202F is localized in transmembrane domain 4 exon7. SIFT and Polyphen2 predicted it was probably damaging. However, this missense mutation was lack of functional data (Li, Zhang, Guo, Tang, & Jiao, 2019).

In 2009, Professor Wang's group described a probably damaging mutation in exon 5, Gln222Leu (NM_000021.4:c.665A>T, NP_000012.1:p.Gln222leu), in a patient with positive family history of EOAD, which caused a glutamine‐to‐leucine substitution of amino acid (Wang et al., 2019). However, no functional data were reported in this mutation. PolyPhen‐2 and SIFT scores suggested that Gln222Leu was probably pathogenic.

The *PSEN1* Leu226Arg (NM_000021.4:c.677T>G, NP_000012.1:p.Leu226Arg) is localized in transmembrane domain 5 exon 7 of PSEN1 protein (Ma et al., 2019). L226R encoded by codon 226 has been reported four times previously (Bagyinszky, Park, et al., 2016; Bagyinszky, Youn, et al., 2016; Coleman, Kurlan, Crook, Werner, & Hardy, 2004; Żekanowski et al., 2006). According to Dr Zhang's group result, L226R mutation may result in substantial changes on the surface of the transmembrane domain of PSEN1.

Mutation Met233Leu (NM_000021.4:c.697A>C, NP_000012.1:p.Met233Leu) of PSEN1 was previously described in patients with AD and detected in China EOFAD in 2015 (Jiang et al., 2015). It maps to the fifth transmembrane domain of PSEN1 on the a‐helix surface in exon 7. An evolutionary conservation analysis revealed that M233L mutation led to a highly conserved amino acid change. The codon 233 of *PSEN1* seems to be a hot spot for mutation, as three other mutations at codon 233 (M233V, M233I and M233T) have been reported. All these mutations were described as pathogenic mutations according to the AD and FTD database. However, the pathogenic mechanism of this mutation remains unknown and needs further investigation.

In 2019, three variants on the exon 7 of *PSEN1* were described by Li, Yang, et al. (2019)). Tyr256Asn (NM_000021.4:c.766T>A, NP_000012.1:p.Tyr256Asn), leading to a tyrosine to asparagine substitution, was found in the first family. Another novel mutation, His214Arg (NM_000021.4:c.641A>G, NP_000012.1:p.His214Arg), was revealed in a patient with positive EOAD family history. And a de novo mutation of *PSEN1* Gly206Val (NM_000021.4:c.617G>T, NP_000012.1:p.Gly206Val) was identified in a patient with very early‐onset sporadic AD. The Gly206Val mutation has been reported previously in one autosomal dominant EOAD family with four affected patients in three generations (Goldman, Reed, Gearhart, Kramer, & Miller, 2002). Based on computational methods, the mutations were all predicted to be deleterious (Li, Yang, et al., 2019; Yang et al., 2016).

The Arg352Cys (NM_000021.4:c.1054C>T, NP_000012.1:p.Arg352Cys) is a previously unidentified *PSEN1* mutation. Arg352Cys in exon 10 is located in the cytoplasmic loop of PSEN1. Arg352Cys altered a relative conserved arginine to cysteine. A subsequent research revealed that this mutation did not increase absolute Aβ42 levels, but instead acted as dominant‐negative presenilin, decreasing Aβ production (Jiang et al., 2015). The pathogenic mechanism of mutation R352C remains unknown and needs further investigation.

Both Dr. Wang and Dr. Cao's groups have revealed a heterozygous point mutation Gly378Glu (NM_000021.4:c.1133G>A, NP_000012.1:p.Gly378Glu). Nucleobase transformed from G to A at position 1133 of exon 11 (Cao, Qiu, Zheng, Lin, & Wang, 2014; Wang, Hao, et al., 2017), leading to a glycine to glutamate at codon 378 of *PSEN1* in two families. This amino acid was encoded across a splice junction and was highly conserved both within the protein and across other species. The result of PolyPhen‐2 showed that Gly378Glu mutation is probably damaging with a score of 1.000 (Lv et al., 2018).

The Phe386Ile (NM_000021.4:c.1156T>A, NP_000012.1:p.Phe386Ile) mutation results in substitution of the amino acid phenylalanine by isoleucine at codon 386. The mutation is located at a position that corresponds to part of the seventh transmembrane domain of PSEN1 (Shea et al., 2017). A vitro study has noted that Phe386Ile increases the level of Aβ42 and decreases the level of Aβ40.

The identified Phe388Leu (NM_000021.4:c.1164C>G, NP_000012.1:p.Phe388Leu) mutation occurs in exon 11 of the *PSEN1* gene, resulting on the seventh transmembrane domain (from 381 amino acid to 401 amino acid) of the PSEN1 protein. Overexpressed PSEN1 F388L mutant in cell culture, we found that F388L mutant‐induced Aβ42 secretion was significantly higher than that of wild‐type PSEN1, resulting in a markedly increased Aβ42/Aβ40 ratio (Zhan et al., 2017).

In 2019, Dr Jia's group identified novel heterozygous mutation Ala434Thr (NM_000021.4:c.1300G>A, NP_000012.1:p.Ala434Thr) in a EOFAD male patient. To picture the role of this mutation, we evaluated its effect of PSEN1 endoproteolysis in SH‐SY5Y cells. The results demonstrated that P433S suppressed PSEN1 endoproteolysis. This might be cause by the distinct codon's position of P433S. Proline‐alanine‐leucine‐proline amino acids, encoded by 433 to 436 codons, is vital for gamma‐secretase activity. The mutation also increased Aβ42, Aβ43, Aβ42/40 ratio (Shen et al., 2019). Also, the same group revealed Ile249Leu (NM_000021.4:c.745A>C, NP_000012.1:p.Ile249Leu) in AD, which was previously described in a sporadic amyotrophic lateral sclerosis case. It could increase Aβ42, Aβ42/40 ratio (Shen et al., 2019).

### 3.5 Mutations in PSEN2

*PSEN2* gene is located on chromosome 1q31‐q42, and it is very similar in structure and function to *PSEN1* (Ridge, Ebbert, & Kauwe, 2013). PSEN2 is a main component of the γ‐secretase complex along with PSEN1, nicastrin, Aph‐1, and PEN‐2 (Wakabayashi & De Strooper, 2008). *PSEN2* mutation alters the γ‐secretase activity and leads to elevation of Aβ42/40 ratio in similar manner to the *PSEN1* mutation. Although PSEN2 shows close homology to PSEN1, less amyloid peptide is produced by PSEN2. Neuritic plaque formation and neurofibrillary tangle accumulation may be seen as neuropathological changes in some people with *PSEN2* mutations (Giri et al., 2016). Park et al. (2012) demonstrated that β‐secretase activity is enhanced by *PSEN2* mutation, through reactive oxygen species‐dependent activation of extracellular signal‐regulated kinase. *PSEN2* mutations are very rare, and to date 45 pathogenic *PSEN2* mutations have been detected worldwide. Till now, six mutations of *PSEN2* were detected in China (Table 3).

**Table 3:** Mutations of PSEN2 discovered in China

Gene	Protein change	Location in the gene (exon)	Gene mutation	Location in PS2 protein	Was it discovered before?	Functional data	PolyPhen Scores	SIFT scores	Reference
PSEN2	Lys82Arg	4	c.245A>G	N‐term	Yes, in Korea	Not available	1 (probably damaging)	0 (damaging)	Shi et al. (2015)
	Pro123Leu	5	c.368C>T	HL‐1	No	Not available	1 (probably damaging)	0.003 (damaging)	Xia et al. (2015)
	Asn141Tyr	5	c.421A>T	TM‐II	No	Increased Aβ42/40 ratio	0.93 (possible damaging)	0 (damaging)	Niu et al. (2014)
	Val150Met	6	c.448G>A	TM‐II	No	Not available	0.72 (possible damaging)	0 (damaging)	Gao et al. (2019)
	Arg163Cys	6	c.487C>T	HL‐II	No	Not available	1 (probably damaging)	0 (damaging)	Gao et al. (2019)
	Val214Leu	7	c.640G>T	TM‐IV	No	Not available	0.97 (probably damaging)	0.09 (tolerated)	Shi et al. (2015)

In 2014, a novel *PSEN2* mutation, Asn141Tyr (NM_000447.3:c.421A>T, NP_000438.2:p.Asn141Tyr), was discovered in a Han Chinese family (Niu et al., 2014). The mutation was identified in two affected family members, who were clinically diagnosed with EOAD. The location of mutation is similar to the Volga‐German mutation (Asn141Ile), and this asparagine to tyrosine change might affect the Aβ42/Aβ40 ratio.

Pro123Leu (NM_000447.3:c.368C>T, NP_000438.2:p.Pro123Leu) was found in a Chinese family in 2015, where the pedigree revealed several affected family members over four generations (Xia et al., 2015). Target region capture sequencing yielded a novel missense mutation at codon 123 which is a heterozygous C to T point mutation at position 368 (c.368C>T) in exon 5 of *PSEN2* leading to a proline‐to‐leucine substitution. According to SIFT and PolyPhen‐2 scores, this mutation is probably pathogenic.

Later, additional mutations have been described in Chinese patients in 2015. The *PSEN2* mutations Val214Leu (NM_000447.3:c.640G>T, NP_000438.2:p.Val214Leu) and Lys82Arg (NM_000447.3:c.245A>G, NP_000438.2:p.Lys82Arg) were identified in two EOAD patients and 1 EOAD patient, respectively (Shi et al., 2015). However, these missense mutations were lack of functional data.

Recently, two novel variants of *PSEN2*, Val150Met (NM_000447.3:c.448G>A, NP_000438.2:p.Val150Met) and Arg163Cys (NM_000447.3:c.487C>T, NP_000438.2:p.Arg163Cys) are described as probably causative mutation according to Dr. Gao's study. Sanger sequencing was performed in this study based on the largest number of AD family sample in Asia (Gao et al., 2019).

In summary, we are the first to summarize all the findings of causative genes (*APP*,* PSEN1* and *PSEN2)* with EOFAD in China. Compared to gene mutations frequently found in Caucasian, we could conclude that most of the variants discovered in Chinese families, are novel mutations, which most of them have not been described in other population.

### 3.6 Clinical features of EOFAD causative genes in China

Most EOFAD cases have insidious onset episodic memory symptoms followed by other cognitive impairments similar to sporadic AD. However, EOFAD has distinctive features including early age at onset (AAO) as well as noncognitive neurological symptoms and a more aggressive course (Mendez, 2019; Wu et al., 2012).

Age at onset is a robust clinical feature of EOFAD compared to the sporadic AD. One study demonstrated that the mean AAO of EOFAD was 50 years with the age ranging from 30 to 65 years (Lv et al., 2018). In general, the AAO of the autosomal‐dominant cases is earlier than that of the familial nonautosomal‐dominant cases. In addition, pedigrees with *PSEN1* mutations have an earlier mean AAO compared to those with *APP* or *PSEN2* mutations (Wu et al., 2012). APOE ε4 alleles have an effect on the AAO in families with *APP* and *PSEN2* mutations, whereas no effect on families carrying *PSEN1* mutations (Wijsman et al., 2005).

Early‐onset familial AD is thought to have a more aggressive course than late‐onset sporadic AD, and EOFAD is associated with faster cognitive decline and higher mortality. In addition to memory impairment, patients with EOFAD often present with prominent cognitive impairment in other domains, such as apraxia, aphasia, or decline in executive ability. It has also been described EOFAD patients may have myoclonus, seizures, spastic paraparesis, and extrapyramidal signs more frequently than sporadic AD (Mendez, 2019).

### 3.7 Clinical features according to genotype

The clinical features of EOFAD are heterogeneous, most probably due to different genetic mutations and epigenetic factors. We summarize the clinical features according to different genotypes in the following section. We also list the numbers of affected families and subjects with each mutation in Table S1.

### 3.8 Clinical spectrum of APP mutations in China

Dr. Huang's study shows the main clinical features of progressive memory impairment followed by rapid progression to severe dementia within 5–10 years with an AAO of around 46–55 years in Taiwanese patients with the novel *APP* Asp678His mutation. In these two large families, there are ten and six symptomatic patients respectively. In addition, cerebral amyloid angiopathy with microhemorrhages were noted in two of symptomatic patients. The characteristic results of brain ^18^F‐AV‐45 PET included the highest standard uptake value ratio in the occipital and cerebellar cortical areas in the genetically positive patients (Huang et al., 2019).

The prominent symptom of the two families carrying Lys687Gln mutation was memory impairment. Both families had three symptomatic patients. In Family A, the index patient carrying this variant presented with progressive memory decline at 63 years of age. Additional symptoms were visual spatial dysfunction and dyscalculia. However, the other two symptomatic patients were unavailable for genetic evaluation. In family B, two members, the index patient (AAO, 52) and his elder brother (AAO, 54), carried Lys687Gln mutation. They had similar symptoms of progressive memory impairment (Jiang et al., 2019).

Val715Met mutation was detected in two patients in an EOFAD pedigree. Both of them developed an insidious onset of difficulties in memory at 40 s (Nan et al., 2011). The mean onset age was 45 years and disease duration was 3 years. One showed irritability and the other presented bradykinesia. Progressive diffuse cortical atrophy in bilateral temporal cortex was observed.

Ile716Thr mutation was revealed in an EOFAD family in Shandong Province in 2017 (Wang, Qin, et al., 2017). There are six symptomatic patients in this pedigree. Their clinical manifestations are marked situational memory impairment, which leads to general cognitive impairment and impaired ability of daily living. Generally, the disease progresses rapidly. The onset age of all patients was ranging from 35 to 40 years old. The first symptom was memory decline and the symptoms progressed rapidly. Abnormal mental behavior and increased muscular tension gradually appeared. Within 5 years, they developed aphasia and loss of daily living ability.

Dr. Jia's group is the first to report the *APP* V717I mutation in five Chinese families (Zhang et al., 2017). The total number of symptomatic patients in these five family is 29. The mean AAO was 54.7 years, mean age at death was 67.1 years, and mean disease duration was 11.7 years. In four of five Chinese families, the initial clinical symptoms were executive dysfunction, disorientation, and subtle memory loss. Only one family initially presented with typical episodic memory impairment. The late onset neurological symptoms were marked spastic paraparesis and cerebellar ataxia. The AAO and the late onset neurological symptoms of Chinese pedigrees were similar to most Caucasian families, whereas the initial symptom was distinct among Chinese patients.

Dr Jia's group also described a novel *APP* Met722Lys mutation Chinese AD pedigree. This Chinese EOFAD pedigree consisted of five symptomatic patients over three generations in which AD was inherited in each generation. The mean AAO of Met722Lys mutation was 42.8 years, mean age at death was 58.9 years, and mean disease duration was 16.1 years. A neuropsychological examination revealed deficits in short‐term memory, long‐term memory, executive function, and orientation, in addition to aphasia (Wang et al., 2015).

The EOFAD with Lys724Met mutation was detected in a family of five affected individuals in two generations from the Hebei Province in Northern China. The mean AAO of *APP* Lys724Met mutation was 46.6 years, mean age at death was 53.4 years, and mean disease duration was 6.8 years. Neuropsychological examination confirmed the presence of verbalization as well as executive dysfunction. Behavioral symptoms consisted of sluggishness and apathy (Peng et al., 2014).

No data are available on the age of onset or on the clinical phenotypes of disease in mutations Val710Gly, IIe718Leu, and Leu720Ser.

In summary, the clinical phenotype of *APP* is heterogeneous among the various mutation. The mean AAO ranged from the 40 s to 50 s. Clinically, patients are mainly characterized by cognitive dysfunction, especially executive dysfunction and disorientation. Extrapyramidal signs, behavioral, and psychiatric symptoms could also be detected in Chinese *APP* EOFAD mutations (Table 4).

**Table 4:** Clinical spectrum of APP mutations of EOFAD in China

Gene	Mutation	Age at onset	Age at death	Disease duration	Neurological symptoms		Behavioral and psychiatric symptoms
					Cognitive symptoms	Noncognitive symptoms	
APP	Asp678His	50.6	58.5	7.9	Progressive memory decline	—	Restlessness, persecutory delusions, self‐talking and slurred speech
	Lys687Gln	56.3	—	—	Progressive memory decline, visual spatial dysfunction, and dyscalculia	—	—
	Val715Met	45	48	3	Memory decline	Bradykinesia	Irritability
	Ile716Thr	35‐40	—	—	Progressive memory decline	Aphasia and increased muscular tension	—
	Val717Ile	54.7	67.1	11.7	Executive dysfunction and disorientation	Spastic paraparesis and cerebellar ataxia	—
	Met722Lys	42.8	58.9	16.1	Memory decline, executive dysfunction, and disorientation	Aphasia	Depression
	Lys724Met	46.6	53.4	6.8	Verbalization and executive dysfunction	Sluggishness and apathy	—

Abbreviation: EOFAD, early‐onset familial Alzheimer's disease.

### 3.9 Clinical spectrum of* PSEN1* mutations in China

Dr Jia's group described *PSEN1* Val97Leu in a large Chinese EOFAD pedigree with 4 symptomatic patients for the first time in 2005 (Jia et al., 2005). The proband's mother passed away and all the other three patients in the FAD family carried this missense mutation. The proband first had memory and counting difficulties, with a noticed irritability, at age 58. These symptoms progressively worsened. For 1 year, she had disturbed orientation and inability to care for herself. Neurology examination revealed impaired memory, disorientation, dyscalculia, agnosia, and apraxia. Patient 2 and patient 3 developed memory disturbance, dyscalculia, apathy, and personality change at age 47 and 46, respectively. Cranial magnetic resonance imaging (MRI) of patient 3 revealed bilateral temporal atrophy and enlarged lateral ventricles.

Phe105Cys was discovered in a Chinese patient, who showed memory impairment at the age of 59 years (Jiao et al., 2014). There are 3 symptomatic patients in the pedigree. Her sibling carried the same mutation, and the dementia was fully developed in her father (died at the age of 60 years). Patients showed typical amnestic symptoms. Myoclonus and seizures were also detected in the patients.

Phe105Leu was detected in an EOFAD family with four affected patients. The proband presented with memory decline at the age of 60 and developed disorientation later. Her twins sister and her brother developed similar symptoms at around 60 years old (Wei et al., 2018).

Gly111Val was identified in a male patient (AAO, 54 years) for the first time. He came to memory clinic 1 year after suffering from memory decline. In the following 2 years, he developed progressive memory decline with executive dysfunction. Bilateral hippocampal atrophy was found in MRI at 57 years of age. Two symptomatic patients were identified in his family. His mother developed similar symptoms with visuospatial impairment at 62 years of age, and finally presented as dementia at about 70 years old (Qiu et al., 2020).

Glu116Lys was detected in a family with seven symptomatic patients spanning four generations. The proband first started seizures at age 12. Memory decline and disorientation showed up at his age of 35. His grandmother, father and father's four siblings presented with similar memory loss with psychiatric symptoms and died at approximately 40 years of age (Li et al., 2016).

Met139Leu mutation was described in a patient who had strongly positive family history of dementia with 11 symptomatic patients over three generations. Age of onset of the proband was 45 years old. Main symptoms in this pedigree were progressive memory decline, with visual spatial dysfunction and irritability (Qiu et al., 2019).

The patient with the *PSEN1* His163Arg mutation was a 45‐year‐old male, who started experiencing memory loss at 42 years of age. There are two symptomatic patients in this family. The other was his father, who developed dementia at 50 years of age, and died at 60 years of age (Shi et al., 2015).

Ile167del was initially diagnosed in a Chinese female patient, for whom the memory loss started at the age of 38 years. Later, she developed personality changes, behavior variants, spastic paraparesis, and disorientation. Spastic paraparesis of this “variant AD” is characterized by insidiously progressive impaired gait and mild weakness in the lower limbs, with the signs of hyperreflexia and clonus. Family history was positive, since four symptomatic members were found. Her mother died at 55 with AD. Her two siblings were also positive for this mutation, and showed similar symptoms (Jiao et al., 2014).

Ser169del was described in a Chinese family with EOAD. This family had four affected individuals in two generations from Southern China (Guo et al., 2010). Initial symptoms were early memory impairment starting between 42 and 50 years. This mutation produced early memory impairment followed by progressive, diffuse cognitive dysfunction, memory decline, asemasia, apraxia, disorientation in time and space, mental aberration, impairment of self‐care after 3–5 years in the course of illness.

Ile202Phe was first reported in an EOFAD pedigree with four symptomatic members. The proband started memory decline at the age of 36 and subsequently developed language disability and personality change. Her siblings carried the mutation showed similar symptoms. However, they did not develop language disability. (Li, Yang, et al., 2019).

Gly206Val was a known mutation, first identified in an American EOAD family in 2002. It affected four patients over three generations, who presented with memory decline at approximately 30 years old, and died in early 40 s (Li, Yang, et al., 2019). In Henan province, a 34‐year‐old male patient with Gly206Val showed progressive memory decline, anxiety, and irritation at the age of 30. Bilateral temporal lobe and hippocampal atrophy were shown in MRI. However, in this case family history was negative (Yang et al., 2016).

A novel mutation, His214Arg, was detected in a 42‐year‐old woman who had a positive family history. She developed memory decline, mood, and behavioral disorders at the age of 41. In this pedigree, nine affected members developed similar symptoms, including her mother, three siblings, and her mother's siblings. Two of her siblings died 10 years after they presented the symptoms (AAO = 40) (Li, Yang, et al., 2019).

The proband carried the Gln222Leu mutation was presented with progressive memory decline, apraxia, visual‐spatial disorders, behavioral, and psychiatric symptoms including temperamental and personality changes. This mutation was also found in another family member (Wang et al., 2019). However, the certain number of affected members was unclear.

Leu226Arg was detected in the proband of an EOFAD family with four symptomatic members. The *PSEN1* Leu226Arg family demonstrated the clinical manifestation of language disability and altered personality at the beginning of the disease (Ma et al., 2019), which differed from the clinical manifestation of the initial memory loss in other *PSEN1* variants in Han Chinese families (Dong et al., 2017; Jiang et al., 2015; Zhan et al., 2017). ^18^F‐FDG‐PET showed hypometabolism in frontotemporal regions, parietal regions, hippocampal areas. Moreover, *PSEN1* variants encoded by codon 226 has been reported four times previously (Bagyinszky, Park, et al., 2016; Bagyinszky, Youn, et al., 2016; Coleman et al., 2004; Gomez‐Tortosa et al., 2010; Żekanowski et al., 2006). The other three Leu226Phe families showed an early‐onset (33–37 years), AD or FTD‐like (frontotemporal dementia) symptoms, and biopsy‐proved AD (Bagyinszky, Park, et al., 2016; Bagyinszky, Youn, et al., 2016). Whereas, the Leu226Arg family in this study had a later age of onset and no Parkinsonism‐like syndrome was observed during the disease. The clinical differences could be ascribed to different ethnicities and environmental factors.

Mutation Met233Leu was identified in an EOFAD family with five symptomatic members. was associated with prominent very early onset at early 40 s. It showed rapidly progressive dementia. The patient became bedridden within three to five years. The mutation was also associated with other neurologic symptoms such as epilepsy and paralysis (Jiang et al., 2015).

Leu248Pro was identified in a 47‐year‐old female patient who started demonstrating progressive memory deficits at the age of 42 years (Jiao et al., 2014). There are five symptomatic members in this family. Her father was clinically diagnosed with AD and died at the age of 69 years. A similar age of onset and phenotype were also reported for her younger brother, who carried the same mutation as the proband. All the patients suffered seizures during the disease process.

At the age of 54, the proband with Ile249Leu presented with progressive memory deficiency, disorientation, and mental and behavioral symptoms including personality change, apathy, social withdraw, and obsessive behaviors. Her cranial MRI showed atrophies in hippocampal and cortical. There were two symptomatic members in this family. Her mother had the same AAO, similar memory impairment, subsequently developed hallucinations and died from AD at the age of 71 (Shen et al., 2019).

Tyr256Asn was recently discovered in a 45‐year‐old woman who started lags in response and short‐term memory deficits at the age of 40. Then she presented with progressive memory impairment, disorientation, confusion, and dyscalculia. At the age of 42, She developed speech disorder, hypermyotonia, static tremor, kinetic tremor, and generalized tonic‐clonic seizures. There were five symptomatic members in this pedigree. Her three siblings and her mother had similar AAO and developed similar memory decline (Li, Yang, et al., 2019).

Arg352Cys was associated with a progressive dementia with an average onset age of 58.8. There were three symptomatic members. This mutation showed a chronic disease course. The patients showed progressive, diffuse memory decline, apraxia, disorientation in the course of illness. Other phenotypes, such as generalized myoclonic jerks, seizures, and psychiatric syndrome were also shown (Jiang et al., 2015).

Two index patients with Gly378Glu were reported by two group in 2014 and 2017. Interestingly, the two family were described with similar AAO and clinical manifestations. Both the two families had nine symptomatic members. The mean AAO of the first EOFAD family was 35 years old. The proband's mother suffered for 5 years and passed away at the age of 40. All of them developed similar cognitive disorder (Cao et al., 2014). In the second pedigree, the average AAO was 34 and the prominent symptom was progressive memory decline. Nine of them had clinical manifestations or the MRI changes of EOFAD (Wang, Hao, et al., 2017).

Phe386Ile mutation was identified in an EOFAD family with six symptomatic members. The proband presented with progressive cognitive decline at 51 years old. He initially developed amnesia, and subsequently developed spatial disorientation, apathy, and inability to handle banking. He developed progressive deterioration with dysphasia and dependency in basic activities of daily living. MRI brain showed bilateral hippocampal atrophy (Shea et al., 2017).

The index patient of Phe388Leu developed progressive memory impairment at the age of 42. Neuropsychological assessment demonstrated that she particularly performed poor in immediate, delayed memory. She also developed psychosis and paranoid delusions. The patient had a strong family history, since four members developed dementia in the family. Her father and two of her siblings developed memory impairment at their early 40 s. The average age of onset of this family was 43 years (Zhan et al., 2017).

*PSEN1* Pro433Ser mutation was detected in a 39‐year‐old male (AAO, 34) for the first time. He has strongly positive family history, since there were seven symptomatic family members. The symptoms were progressive memory decline, agitation, depression, and disorientation, impairment of language ability, attention, judging, and problem‐solving ability. His cranial MRI showed atrophies in hippocampal and cortical. Another six patients over three generations in this pedigree had very early age of onset in their 30 s, developed semblable symptoms, subsequently died in their 40 s (Shen et al., 2019).

Ala434Thr was detected in two symptomatic patients of an EOFAD family. The proband's mother was clinically diagnosed with AD at the age of 55 years. Although both of them carried the same mutation, there were some differences in the phenotypes and the course of the disease between them. The prominent symptoms of the proband patient were hallucinations and delusions at the age of 35 years. Progressive memory loss and other cognitive impairments were noted after 3 years. Her mother presented with memory impairment, however, other cognitive domains were preserved (Jiao et al., 2014).

No data are available on the age of onset or on the clinical phenotypes of disease in mutations Val103Gly, Ala136Gly, Phe177Val, and Gly378Glu.

In conclusion, EOFAD with *PSEN1* mutations typically have an AAO in the early 40 s, ranging from 35 to 65 years. Although a subcortical pattern of neuropsychological deficits has been noted in some case reports, the cognitive profile has so far predominantly shown amnestic and involved multiple domains. In addition to cognitive symptoms, EOFAD with *PSEN1* mutations may present with some unusual clinical features, in particular myoclonus and seizures. Despite almost all these symptoms having been reported in sporadic AD, extrapyramidal signs, behavioral, and psychiatric symptoms (anxiety, hallucinations, delusions) and ataxia are significantly more frequently found in EOFAD with *PSEN1* mutations (Table 5).

**Table 5:** Clinical spectrum of PSEN1 mutations of EOFAD in China

Gene	Mutation	Age at onset	Age at death	Disease duration	Neurological symptoms		Behavioral and psychiatric symptoms
					Cognitive symptoms	Noncognitive symptoms	
PSEN1	Val97Leu	36.5	52.4	15.9	Memory disturbance, dyscalculia	—	Apathy and personality change
	Phe105Cys	59	60	—	Amnestic	Seizures	—
	Phe105Leu	60.5	—	—	Memory decline and disorientation	—	—
	Gly111Val	55	—	—	Progressive memory decline and executive dysfunction	—	—
	Glu116Lys	35	40	5	Memory decline and disorientation	—	—
	Met139Leu	51.6	59.2	7.6	Progressive memory decline, and visual spatial dysfunction	—	Irritability
	His163Arg	45	60	15	Memory decline	—	—
	Ile167del	38	55	15.5	Memory decline, disorientation	Spastic paraparesis	Behavior variants
	Ser169del	44.8	49.3	4.5	Memory decline, disorientation	Apraxia, myoclonus, and seizures	—
	Ile202Phe	43	53	10	Memory decline, language disability	—	Depression, irritability
	Gly206Val	30	—	—	Progressive memory decline	—	Anxiety and irritation
	His214Arg	40.5	50	10	Memory decline	—	—
	Gln222leu	—	—	—	Progressive memory loss, visual‐spatial disorders, and apraxia	—	Psychobehavioral disorders, temperamental, and personality changes.
	Leu226Arg	60	—	—	Language disability, memory decline	—	Personality change, irritability
	Met233Leu	40.6	44.8	4.2	Progressive memory decline	Seizures and paralysis	—
	Leu248Pro	42	69	11	Progressive memory decline	No	—
	Ile249Leu	54	71	17	progressive memory decline, and disorientation	—	Personality change, apathy, social withdraw, and obsessive behaviors
	Tyr256Asn	40	46	7	Memory decline, disorientation, and dyscalculia	Hypermyotonia, static and kinetic tremor, seizures	—
	Arg352Cys	58.8	69.5	10.7	Progressive memory decline and disorientation	Apraxia, myoclonus, and seizures	Personality changes
	Gly378Glu	34.5	40.5	5	Progressive memory decline, language disability	—	—
	Phe386Ile	57	—	—	Amnesia and difficulty working	Seizures	—
	Phe388Leu	43	44.4	1.41	Progressive memory decline	Psychosis and paranoid delusions	—
	Pro433Ser	34.5	47	12	Progressive memory decline, disorientation, impairment of language, attention, judging, and problem solving	—	Agitation, depression
	Ala434Thr	35	55	8	Progressive memory decline	—	Hallucinations and delusions

Abbreviation: EOFAD, early‐onset familial Alzheimer's disease.

### 3.10 Clinical spectrum of PSEN2 mutations in China

In 2014, a novel *PSEN2* mutation Asn141Tyr, was discovered in a Han Chinese family. six patients developed dementia in this family (Pei et al., 2014; Wei et al., 2018). The proband developed progressive memory impairment with the inability to handle financial matters and find personal items at the age of 43 years. Several family members died with dementia. Her sister developed memory decline at the age of 49 years and later developed speech difficulties and disorientation. In the later disease stages, she developed paranoia, visual hallucinations, and agitation.

Pro123Leu was found in a Chinese family, where the pedigree had four affected family members over four generations (Bagyinszky, Park, et al., 2016; Cao et al., 2014; Coleman et al., 2004; Wei et al., 2018; Żekanowski et al., 2006). At the proband patient, disease started at the age of 57 years, with personality changes and memory problems. ^18^F‐FDG‐PET shows hypometabolism in right temporoparietal and precuneus. Cognitive impairment with parkinsonism and myoclonic jerks were quite common phenotypes among the patients. However, mutation did not segregate with the disease, since several asymptomatic relatives also carried the mutation.

Later, additional mutations have been described in Chinese patients. Val214Leu was found in two AD patients from two pedigrees. No detailed information was provided in family A. While family B had two affected patients. The prominent symptoms of the proband was progressive memory loss. PIB PET showed amyloid deposits on bilateral frontal, lateral temporal, and parietal lobes, cingulate cortex, precuneus, and striatum. ^18^F‐FDG‐PET showed hypometabolism on left frontal lobe, parietal lobe, bilateral temporal lobe, insular lobe, cingulate cortex, precuneus, caudate nucleus, and thalamus. Lys82Arg was also found in a female patient. No detailed family information was provided. She developed AD with depression and language impairment at the age of 50 years. Atrophy was observed in the posterior region and hippocampus, and PIB PET showed amyloid deposits on the bilateral frontal lobe, lateral temporal lobe, parietal lobe, posterior cingulate cortex, precuneus, and striatum (Shi et al., 2015). ^18^F‐FDG‐PET showed hypometabolism on the bilateral temporo‐parieto‐occipital adjunction area, posterior cingulate cortex, and precuneus.

No data are available on the age of onset or on the clinical phenotypes of disease in mutations Val150Met and Arg163Cys.

Of the three genes known to cause EOFAD, mutations in PSEN2 are the least common, and therefore the literature is relatively scant. Compared to PSEN1 mutation carriers, carriers with PSEN2 mutations have a later AAO, relatively longer disease duration and a more variable disease expression (Table 6).

**Table 6:** Clinical spectrum of PSEN2 mutations of EOFAD in China

Gene	Mutation	Age at onset	Age at death	Disease duration	Neurological symptoms		Behavioral and psychiatric symptoms
					Cognitive symptoms	Noncognitive symptoms	
PSEN2	Lys82Arg	50	—	—	—	Speech difficulties	Depression
	Pro123Leu	57	—	—	Memory decline	Parkinsonism and myoclonus	Personality changes
	Asn141Tyr	46	49	16	Memory decline and disorientation	Speech difficulties	Paranoia, visual hallucinations, and agitation
	Val214Leu	60	—	—	Progressive memory decline	—	—

Abbreviation: EOFAD, early‐onset familial Alzheimer's disease.

## 4 CONCLUSIONS

Because the overall population and aging population in China are increasing, genetic testing of patients with AD is important in the diagnosis of dementia. EOFAD is a condition characterized by dementia onset at a relatively young age and a positive family history for dementia. Mutations in APP, PSEN1, and PSEN2 genes are, to date, the only deterministic factors for EOFAD. Thus, studies of patients with EOFAD and their families are of paramount importance to provide the complete clinical course of AD progression. To date, although more than 400 mutations have been identified in EOFAD patients, there remains no systemic investigation and summary on causative gene mutations in China. In this study, 10 *APP* mutations, 27 *PSEN1* mutations and six *PSEN2* mutations were identified and summarized. As far as we know, this is the first systemic review of AD causative gene mutations in patients with EOFAD in China.

Compared with the pathogenic variants among Caucasian, most mutations described in China are novel. The proportion of causative mutation in patients with EOFAD was similar of that in the United Kingdom and France, when the sample size was reasonable and whole‐exome sequencing was performed (Jiang et al., 2019). In addition, the symptoms and AAO were similar when the patients shared the same mutation, because of the genotype–phenotype correlation. And the disease duration might be affected by caring situation, medical treatments, environment, and so on, which vary in different countries and families.

In addition, our study expands the clinical phenotype spectrum of presenile familial AD and points to a strong influence of genetic variants in the development of the clinical phenotypes. Clinical diversity might be the result of different genetic mutations that directly or indirectly affect disease progression or protect against the disease. Therefore, our findings, further suggest different clinical phenotypes because of different amino acid transversions. Further studies will be focused on exploring if there is an unidentified mechanism between causative gene mutations and phenotypic heterogeneity.

## CONFLICT OF INTEREST

None declared.

## AUTHORS’ CONTRIBUTIONS

QQ and YW searched and acquired data. QQ and YSY drafted the manuscript. YT and JPJ revised the article. YYL helped to draft the manuscript. All authors read and approved the final manuscript.

## Supporting information

Table S1
