# Supplementary material for: Gene mutations associated with early onset familial Alzheimer’s disease in China: An overview and current status
Source: Mol Genet Genomic Med. 2020 Aug 6;8(10):e1443. doi: 10.1002/mgg3.1443 (PMC7549583; doi:10.1002/mgg3.1443)
Supplement: Supplementary file 1 — Table S1 [file MGG3-8-e1443-s001.docx]

**Supplementary Table 1. Families/subjects affected with each mutation**

| **Gene** | **Mutation** | **Numbers of**  **families** | **Subjects with clinical phenotype** |
| --- | --- | --- | --- |
| APP | Asp678His | 2 | 16 |
|  | Lys687Gln | 2 | 6 |
|  | Val715Met | 1 | 2 |
|  | Ile716Thr | 1 | 6 |
|  | Val717Ile | 1 | 29 |
|  | Met722Lys | 1 | 5 |
|  | Lys724Met | 1 | 5 |
| PSEN1 | Val97Leu | 1 | 4 |
|  | Phe105Cys | 1 | 3 |
|  | Phe105Leu | 1 | 4 |
|  | Gly111Val | 1 | 2 |
|  | Glu116Lys | 1 | 7 |
|  | Met139Leu | 1 | 5 |
|  | His163Arg | 1 | 2 |
|  | Ile167del | 1 | 4 |
|  | Ser169del | 1 | 4 |
|  | Ile202Phe | 1 | 4 |
|  | Gly206Val | 1 | 4 |
|  | His214Arg | 1 | 9 |
|  | Gln222leu | 1 | 2 |
|  | Leu226Phe | 1 | 4 |
|  | Met233Leu | 1 | 5 |
|  | Leu248Pro | 1 | 5 |
|  | Ile249Leu | 1 | 3 |
|  | Tyr256Asn | 1 | 5 |
|  | Arg352Cys | 1 | 3 |
|  | Gly378Glu | 2 | 18 |
|  | Phe386Ile | 1 | 6 |
|  | Phe388Leu | 1 | 4 |
|  | Pro433Ser | 1 | 7 |
|  | Ala434Thr | 1 | 2 |
| PSEN2 | Lys82Arg | 1 | 2 |
|  | Pro123Leu | 1 | 4 |
|  | Val214Leu | 2 | 2 |
|  | Lys82Arg | 1 | 1 |
